# Efficacy of adhesive discs for nocturnal xerostomia after head and neck radiotherapy: a randomized crossover trial

**DOI:** 10.1007/s00784-026-06798-8

**Published:** 2026-02-28

**Authors:** Christoph Matthias Schoppmeier, Stefan Zimmer, Alicia Maria Meyer-Hofmann, Elmar Ludwig, Anna Greta Barbe

**Affiliations:** 1 Faculty of Medicine, Polyclinic for Operative Dentistry and Periodontology, University of Cologne, University Hospital Cologne, Kerpener Str. 32, 50931 Cologne, Germany; 2 Department of Operative and Preventive Dentistry, Faculty of Health, Witten/Herdecke University, Alfred-Herrhausen-Str. 50, 58448 Witten, Germany; 3 Dental group practice Dr. Markus Dirheimer and Dr. Elmar Ludwig, Neue Straße 115, 89073 Ulm, Germany

**Keywords:** Xerostomia, Radiotherapy, Head and Neck Cancer, Mucoadhesive Agents, Salivary Flow

## Abstract

**Objectives:**

Radiotherapy for head and neck cancer (HNC) frequently results in irreversible salivary gland damage and persistent xerostomia. This study aimed to assess the efficacy of a topical mucoadhesive disc containing xylitol in comparison to water with respect to (i) subjective symptom burden of nocturnal dry mouth and (ii) objective salivary flow parameters in post-radiotherapy HNC patients.

**Materials and methods:**

In a randomized, single-blind, crossover-controlled clinical trial, 33 patients with radiotherapy-induced xerostomia received either adhesive discs (XyliMelts) or water across two treatment phases, separated by a washout period of 7 days. Subjective outcomes were assessed using a 100 mm visual analogue scale (VAS) and the Shahdad symptom relief questionnaire. Objective measures included unstimulated and stimulated salivary flow rates. Within-group pre-post comparisons were analyzed using paired t-tests or Wilcoxon signed-rank tests; between-group differences were assessed using independent t-tests or Mann-Whitney U tests (*p* < 0.05).

**Results:**

Adhesive discs use was associated with significantly greater subjective symptom relief (76% vs. 24%; *p* < 0.001), improved sleep continuity (67% vs. 9%; *p* < 0.001), and higher willingness to continue product use (70% vs. 30%; *p* = 0.044). Perceived oral moisture, comfort, and flavor ratings were also higher with adhesive discs. No changes were observed in salivary flow parameters. Tolerability was high, with minimal adverse events reported.

**Conclusions:**

Topical, symptom-oriented interventions may provide meaningful relief for patients suffering from post-radiotherapy xerostomia, especially when regenerative treatment options are limited. Further clinical trials are warranted to confirm these findings in larger populations and to evaluate long-term efficacy and safety.

**Clinical relevance:**

Mucoadhesive xylitol discs appear to be well tolerated and are perceived as beneficial by patients experiencing xerostomia following HNC therapy.

**Supplementary Information:**

The online version contains supplementary material available at 10.1007/s00784-026-06798-8.

## Introduction

Dry mouth is a common issue in dentistry, presenting a significant challenge for both patients and healthcare professionals. Xerostomia refers to the subjective sensation of a dry mouth, which can occur even without an objectively measurable reduction in salivary flow. In contrast, hyposalivation can be objectively diagnosed through the measurement of salivary output [1]. Due to demographic changes and the corresponding increase in morbidity, the prevalence of dry mouth, both subjective and objective, is rising steadily, which is largely attributed to the increasing multimorbidity and polypharmacy in the population [2]. According to current estimates, approximately 900,000 new cases of head and neck cancer (HNC) were diagnosed globally in 2020. The annual incidence of HNC is approximately 150,000 cases in Europe and around 60,000 cases in North America, with both regions having some of the highest age-standardized incidence rates worldwide [3]. The two primary risk factors for HNC are tobacco use, particularly in combination with excessive alcohol consumption, which predominantly contributes to tumors of the oral cavity and pharynx, and infections with human papillomavirus (HPV), especially HPV-16, which has increasingly been identified as the leading cause of oropharyngeal tumors [4]. Radiotherapy (RT) is a well-established treatment option for HNC, alongside surgical tumor resection, but it is associated with significant acute and long-term side effects. A major contributing factor to these side effects is the inclusion of salivary glands in the radiation fields, often resulting in their damage [5]. Patients typically receive a radiation dose of 60 to 70 Gy, which frequently leads to at least temporary, and in many cases permanent, impairment of salivary gland function [6]​. This results in symptoms such as oral burning, difficulty speaking and swallowing, severe dry mouth, and an increased risk of caries and oral infections [7]. Additionally, halitosis, depressive symptoms, and sleep disturbances may occur, all of which can severely impair patients’ quality of life [8, 9].

The treatment of RT-induced dry mouth is primarily symptomatic [10]. The most common method of relief is drinking sips of water. However, water is a poor moisturizer for the oral mucosa, as it evaporates relatively quickly [11]. The mucoadhesive xylitol disc; XyliMelts (OraHealth Corporation, Bellevue, WA, USA), an over-the-counter product for alleviating dry mouth (pH 8.1), is recommended for both nighttime and daytime use. Adhesive discs adhere to the oral mucosa, molars or even dental protheses and release 550 mg of xylitol in a controlled manner in a period of 1–4 h (daytime use) or 4–8 h (nighttime use). Xylitol is a non-fermentable carbohydrate that resembles common table sugar in taste. Additionally, the discs contain cellulose gum, chemically modified by the addition of carboxyl, hydroxyl, methyl, and/or propyl groups, along with a mild mint flavor that is gradually released; but a mint-free version (used in this study) is also available [11]. The discs are supposed to stimulate salivary flow mechanically, while xylitol and cellulose gum act as lubricants and moisturizers. Burgess and Lee demonstrated initial positive effects on the subjective perception of xerostomia [11]. In addition, adhesive discs have also shown promising results in patients with Sjögren’s syndrome, indicating their potential efficacy in various xerostomia-related conditions [12]. However, to date, no clinical randomized studies have evaluated the efficacy of this product on both hyposalivation and xerostomia in HNC patients with RT-induced dry mouth. Therefore, we conducted a randomized, single-blind, crossover study in this vulnerable patient group. Our aim was to assess the efficacy of adhesive discs compared to water in terms of subjective burden from RT-induced dry mouth and various objective clinical parameters.

The null hypotheses of this study were as follows:

1. There is no difference in subjective symptom relief of xerostomia (measured by a 100 mm visual analog scale [VAS]) in patients with nocturnal dry mouth after head and neck radiotherapy, both in pre- and post-treatment comparisons and compared to the use of water.
2. There is no difference in the objective improvement of salivary flow rates (unstimulated and stimulated) between the use of adhesive discs and water in patients with RT-induced dry mouth.

## Materials and methods

### Ethics

The study was approved by the local ethics committee of Witten/Herdecke University (reference number 118/2022, approval date: August 18, 2022). Additionally, the study was registered in the German Clinical Trials Register (DRKS) (DRKS00009685; (https://www.germanctr.de)). The preparation of this investigation followed the protocol established via the Consolidated Standards of Reporting Trials statement (CONSORT) [13].

### Subjects

**Fig. 1:**
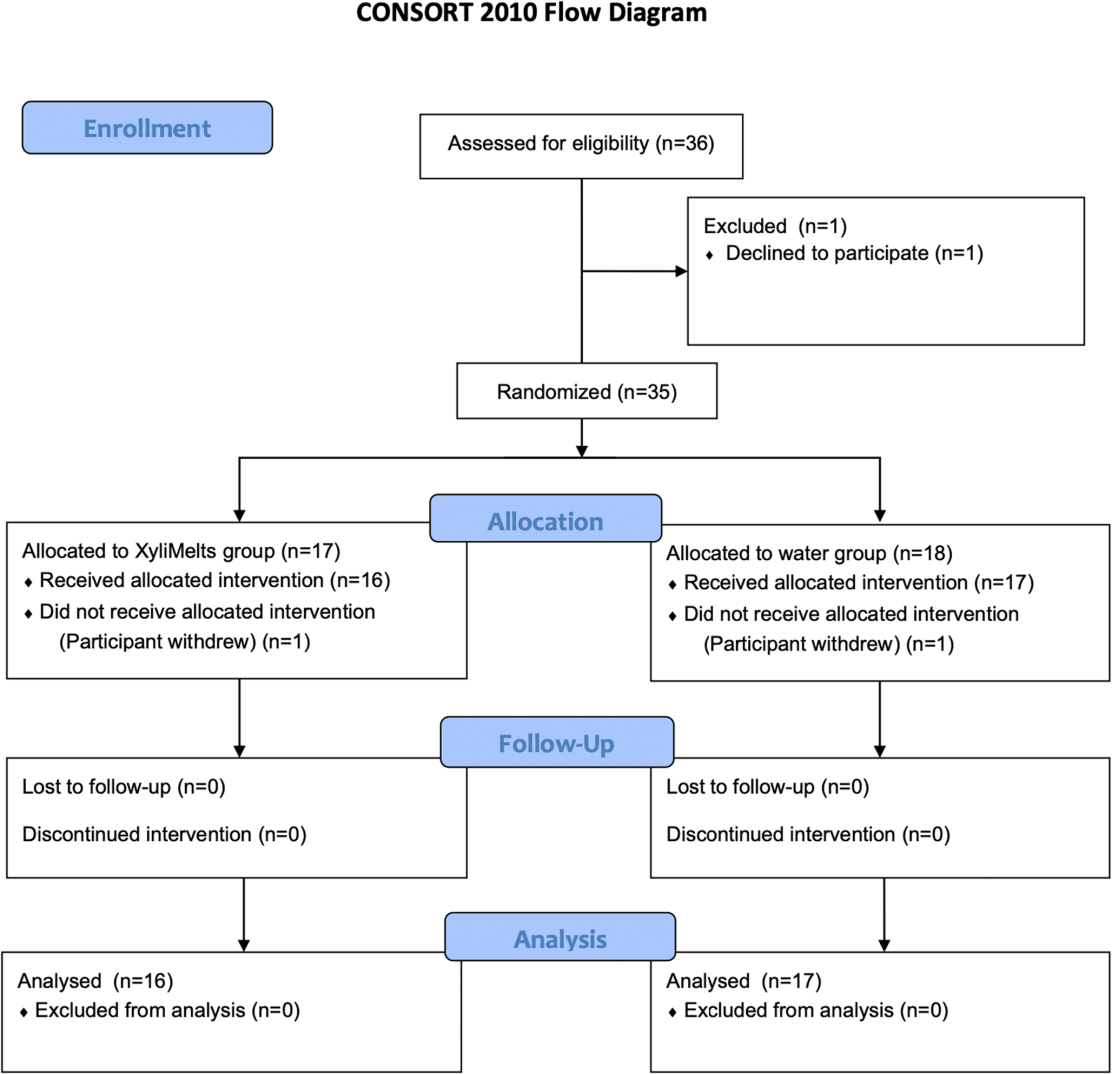
Note. This data is mandatory. Please provide

A randomized, single-blind crossover study with *n* = 36 included participants, who reported subjective symptoms of dry mouth, was conducted (Fig. 1).

Patient recruitment took place between October 2023 and September 2024. Patients were recruited from the dental office of Elmar Ludwig (EL) in Ulm, Germany. All patients who met the eligibility criteria and provided informed consent were included in the study. Participants were initially screened for predetermined inclusion and exclusion criteria.

### Inclusion/Exclusion criteria

Specific inclusion and exclusion criteria were established to ensure a homogeneous study population and to minimize potential confounders. The inclusion criteria required participants to be at least 18 years of age and to have provided written consent for study participation. Additionally, subjects had to suffer from subjective xerostomia, with a visual analogue scale (VAS) score of at least 40 out of 100, assessed immediately upon waking. Patients were asked to respond to the question “Please rate the severity of your dry mouth” by marking their answer on a 100-mm visual analogue scale ranging from 0 = no dry mouth at all to 100 = worst imaginable dry mouth. All participants must have undergone radiotherapy for head and neck cancer lasting at least six weeks. To ensure symptom stability, the primary treatment must have been completed at least 3 months before the study’s commencement.

Exclusion criteria included an existing or planned pregnancy during the study period, ongoing radiotherapy, chemotherapy, or antibody therapy. Furthermore, individuals with acute inflammatory oral conditions, such as an abscess, were excluded, as were those who experienced changes in medication that could potentially affect salivary flow during the study.

### Study design and procedures

The investigational product, XyliMelts (containing no cariogenic ingredients), was administered as a mucoadhesive disc applied in the oral vestibule before bedtime. Participants used one disc per night at the time of going to sleep, as documented in the study diary, and placed it in the buccal vestibule in the molar region. One participant reported minor initial discomfort; no difficulties with disc retention were reported. The comparator intervention consisted of commercially available non-carbonated mineral water (Evian; Danone Deutschland GmbH, Frankfurt am Main, Germany). The water comparator involved still mineral water without a prescribed volume, thereby reflecting natural drinking habits. Each treatment period lasted seven consecutive nights.

### Wash-out period (7 Days)

During the seven-day wash-out period, participants were not permitted to use any other products for hyposalivation or xerostomia. From the first day of the second test phase, participants switched to the alternative study product (crossover).

### Examination appointments

At the start of the study (Baseline 1), a detailed medical history and medication review were conducted. During subsequent visits, patients were asked about relevant new events and changes in medication or dosage. A dental clinical examination and both subjective and objective assessments of dry mouth parameters were performed. These assessments were repeated at the end of the first phase (Follow-up 1), at the start of the second phase (Baseline 2), and at the end of the second phase (Follow-up 2). Participants completed a structured study diary for seven consecutive nights during each study phase to document use of the saliva substitute and nocturnal events. For each night, participants recorded the date, time of going to bed, number of nocturnal awakenings, number of nocturnal fluid intake events, time of getting up in the morning, and any relevant remarks (e.g., alcohol consumption). For analysis, the number of nocturnal awakenings and nocturnal fluid intake events per night were quantified for each participant, and mean values were calculated. No participant reported any nocturnal fluid intake other than water during nights when the adhesive disc was used throughout the study period. Tolerability and possible side effects were also recorded in the diary. In this context, “side effects” refer to any unintended or unpleasant reactions associated with the use of the adhesive discs, such as mild oral irritation, discomfort at the application site, or unfavourable taste sensations. “Tolerability” describes how well patients were able to use the discs without experiencing significant discomfort or difficulties, including their ability to keep the disc in place throughout the night and to continue its use without notable adverse reactions.

### Parameters assessed

Demographic and anthropometric data, along with individual lifestyle factors (e.g., smoking, fluid intake based on self-reported estimates of approximate daily intake in litres per day) and sleep habits and quality, were recorded for each participant.

### Salivary pH

The pH of the saliva was determined using indicator strips (pH Speichel & Urin, BMUT, Berlin, Germany), which displayed pH levels based on color change. The strips were placed on the dorsal surface of the tongue for 1 min prior to each measurement, and participants were instructed to press their tongue against the palate during this time. Immediately after removal, the strips were read out.

### USFR (Unstimulated Salivary Flow Rate)

The method for collecting unstimulated and stimulated salivary flow rates is well documented in the literature [1, 14]. Saliva samples were always taken on the same weekday at the same times, between 9:00 and 11:00 a.m., in a quiet room at EL`s dental office. A single investigator (EL) collected all samples. Participants were instructed not to eat, not to drink, not to smoke and not to practice oral hygiene ninety minutes before the examination. During sampling, participants sat upright, leaned slightly forward, and kept their mouth open without movement, allowing saliva to drip into a disposable cup for 15 min, with the collected volume recorded at 5, 10, and 15 min. The collected saliva was weighed using a precision scale (Joshs MR1, SSR Produkt GmbH, Oldenburg, Germany and the volume (ml/min) was calculated based on a saliva density of 1.01 g/ml. A salivary flow rate of less than 0.1 ml/min was considered indicative of objective hyposalivation [15].

### VAS for xerostomia

The questionnaire included a continuous visual analogue scale (VAS), ranging from 0 mm (completely normal saliva) to 100 mm (no saliva at all), following the methodology described by Dirix et al. [8]. The exact questions were: “To what extent do you suffer from dry mouth in general?” and “To what extent do you suffer from dry mouth at night?“. In addition, the questionnaire included an item asking participants to estimate how often they wake up at night due to dry mouth on average. This latter question was developed by the authors and was not part of the original questionnaire reported by Dirix et al.

### Symptom relief questionnaire

Subjects completed a dichotomous questionnaire during the first visit and after each application regarding their individual symptom relief, based on the questionnaire by Shahdad et al. [10]. All questions are presented in Table 1.

**Table 1:** After-use questionnaire for patient-related outcome measures after 1 week use of water or XyliMelts, respectively. The questions were based on the questionnaire by Shahdad et al. [10]

	Yes/No	All	Water	Xylimelts	*p*
			N ^*a*^ (%)	N ^*a*^ (%)	
Did the product make your dry mouth better?	N	32	25	7	**< 0.001**
	Y	33	8	25	
Did the product make chewing easier?	N	45	24	21	0.708
	Y	11	5	6	
Did the product make swallowing easier?	N	43	23	20	0.201
	Y	20	10	10	
Did the product make talking easier?	N	51	25	26	0.922
	Y	7	4	3	
Did the product improve the sensation of taste?	N	54	26	28	0.388
	Y	1	0	1	
If you have a burning mouth, did the product improve the burning sensation?	N	18	11	7	0.406
	Y	19	10	9	
Did the product reduce any bad taste that you get in your mouth?	N	46	24	22	0.631
	Y	7	4	3	
Did the product stop you waking in the night?	N	36	26	10	**< 0.001**
	Y	25	3	22	
Would you like to continue using this product?	N	18	13	5	**0.044**
	Y	37	14	23	
If your wear dentures, did the product help with the retention of the dentures?	N	12	7	5	0.561
	Y	43	21	22	
Did the product improve your quality of life?	N	31	20	11	**0.008**
	Y	27	8	19	
Did you visit people more than you used to?	N	48	25	23	0.580
	Y	1	1	0	
Did you speak to people more than you used to?	N	51	27	24	0.855
	Y	5	2	3	
Did you get out of the house more than you used to?	N	53	28	24	0.570
	Y	3	1	2	
Was the product easy to use?	N	1	1	0	0.529
	Y	60	30	30	
Did you feel better for using this product?	N	30	22	8	**0.002**
	Y	26	7	19	

### SFR (Stimulated Salivary Flow Rate)

Salivary stimulation was initiated by having participants rinse their mouth, followed by chewing on 1 g of paraffin wax pellets (Paraffin Pellets, Aurosan, Essen, Germany). Stimulated saliva was then collected into sterile cups at 60-second intervals over a total period of 5/10/15 minutes. The collected saliva was weighed using a precision scale (Joshs MR1, SSR Produkt GmbH, Oldenburg, Germany and the volume (ml/min) was calculated based on a saliva density of 1.01 g/ml. A stimulated salivary flow rate below 0.7 ml/min was considered indicative of objective hyposalivation [15].

### Blinding and randomization

This study was conducted in a blinded manner to prevent investigator bias. Participants were thoroughly briefed about the study design and blinding process. They were instructed not to disclose their assigned treatment phase to the investigator. The treatment-package (mint-free adhesive discs, water, diary) was given to the patients by the study dentist. Participants were assigned to one of two treatment groups – either the adhesive discs group or the water group. Randomization was performed using prepared envelopes. Each participant was assigned a study arm. The study dentist distributed the corresponding blinded envelope without knowledge of the treatment group according to the randomized group.

### Sample size calculation

The sample size for this study was determined based on a crossover design. Assuming a two-sided alpha level of 5%, a statistical power of 80%, and a correlation coefficient of ρ = 0.5 between the two treatment periods, a total of 32 participants was calculated to be sufficient. This calculation was based on the methodology described by Shahdad et al. [10]. To account for an anticipated dropout rate of 10%, the initial sample size was increased by 10%, resulting in a planned enrolment of 36 participants.

### Data analysis

Data were analyzed using SPSS Version 29.0.1 (IBM Corp., Armonk, NY, USA). Descriptive statistics were calculated for all continuous variables and are presented as means with standard deviations (SD) for normally distributed data, or medians with interquartile ranges (IQR) where appropriate. Categorical data are expressed as absolute frequencies and percentages. Normality of distribution was assessed using the Kolmogorov-Smirnov-test. For comparisons between baseline and post-intervention values within groups, the paired t-test was used for normally distributed data, and the Wilcoxon signed-rank test for non-parametric data. Differences between the adhesive discs and water groups were analyzed using the independent samples t-test for continuous variables or the Mann-Whitney U test when assumptions of normality were not met. Categorical variables were compared using the Chi-squared test or Fisher’s exact test, as appropriate. All comparisons between mucoadhesive discs and water conditions for the diary answer analysis were conducted using two-sided paired t tests, reflecting the within-subject study design. A two-tailed significance level of *p* < 0.05 was considered statistically significant. No corrections for multiple comparisons were applied due to the exploratory nature of the study.

## Results

A total of 33 participants (24% female, mean age females 61.6 ± 8.4 years, 76% male; mean age males 64.5 ± 5.3 years) participated throughout the protocol in accordance with the predefined inclusion and exclusion criteria. The mean number of comorbid conditions (excluding cancer) was 2.1 ± 2.2, and participants had 22.1 ± 6.7 remaining teeth. The most common comorbidities were cardiovascular and endocrine disorders, with hypertension and hypothyroidism being the most frequent (each 9/33; 27%), followed by hypercholesterolemia (5/33; 15%) and coronary artery disease (3/33; 9%). Participants had received a mean total radiation dose of 68.3 ± 24.1 Gy, delivered over a mean of 32.6 ± 8.9 fractions and a mean treatment duration of 6.9 ± 2.1 weeks. Details on specific tumor type, TNM staging, radiation doses, number of fractions, and overall treatment duration are provided in Supplement S1.

Baseline salivary characteristics included a mean unstimulated salivary pH of 6.4 ± 0.6, unstimulated salivary flow rate (USFR) of 0.9 ± 0.8 ml/5 min, and stimulated salivary flow rate (SSFR) of 5.4 ± 4.3 ml/5 min. The mean xerostomia score on a 100 mm visual analogue scale (VAS) was 65.0 ± 17.2, and participants reported waking 2.8 ± 1.9 times per night due to dry mouth (Table 2). Participants estimated a mean daily fluid intake of approximately 2.1 ± 0.69 L/day based on self-reported information without differences between groups (*p* > 0.05).

**Table 2:** Baseline characteristics

All (*n* = 33)	Mean (SD) Median range
**Age (years)**	65 ± 6.7 64 54–80
**Number of chronic diseases**	2.1 ± 2.2 2 0–8
**APIs**	3.5 ± 3.6 3 0–14
**Number of teeth**	22.1 ± 6.7 24 4–31
**pH**	6.4 (0.6)
**Unstimulated salivary flow rate (ml/min)**	0.9 (0.8)
**Stimulated salivary flow rates (ml/min)**	5.4 (4.3)
**Xerostomia VAS (0-100)**	65 (17.2)
**Times waking up at night due to dry mouth**	2.8 (1.9)
	N (%)
**Female**	8 (24)
**Male**	25 (76)
**Smoker**	22 (18.2)
**Adjuvant chemotherapy**	22 (67)
**Surgery**	23 (69.7)
**Neck dissection**	22 (66.7)

### Subjective xerostomia symptoms and patient-reported outcomes

Both treatment conditions showed comparable general and nocturnal xerostomia VAS scores (as described in 2.10) at baseline and follow-up. Baseline nighttime dryness was significantly lower in the adhesive discs group compared to water (*p* = 0.026), but this difference was no longer present post-intervention (*p* = 0.258). Nocturnal awakenings due to dry mouth did not differ significantly between groups at baseline (*p* = 0.901) or follow-up (*p* = 0.067), although a trend favoring adhesive discs was observed (Table 3). Post-intervention patient-reported outcomes (as described in 2.10) indicated a markedly greater perceived effectiveness of adhesive discs across multiple domains. Symptom relief was reported by 76% of participants using adhesive discs versus 24% in the water group (*p* < 0.001). Significantly more adhesive discs users reported improved sleep continuity (66.7% vs. 9.1%; *p* < 0.001), willingness to continue use (69.7% vs. 30.3%; *p* = 0.044), and quality of life improvements (57.6% vs. 24.2%; *p* = 0.008). No between-group differences emerged for chewing, swallowing, speaking, taste, burning sensations, or social interaction. On the 100-mm VAS, adhesive discs were rated significantly higher than water regarding perceived effectiveness (median 62.5 vs. 25.0; *p* < 0.001), flavor (70.0 vs. 50.0; *p* = 0.017), oral comfort (65.5 vs. 50.0; *p* = 0.004), and perceived moisture film (75.0 vs. 38.0; *p* < 0.001) (Tables 1 and 4). The mean number of nocturnal awakenings reported in the diary (as described in 2.6) was significantly reduced under mucoadhesive discs compared with water (1.23 ± 1.08 vs. 1.75 ± 1.36; *p* = 0.0039). Likewise, participants reported significantly fewer night-time drinking episodes when using mucoadhesive discs than when drinking water (0.43 ± 0.69 vs. 1.13 ± 1.27; *p* < 0.001).

**Table 3:** Subjective Xerostomia symptoms (VAS 0-100) VAS ranging from 0 mm (completely normal saliva) to 100 mm (no saliva at all), following the methodology described by Dirix et al. [8]

Question	Wasser	Xylimelts	*p*
To what extent you suffer from dry mouth in general?			
Baseline	70 (20/95)	70 (0/95)	0.579
Follow-Up	71 (20/99)	70 (10/100)	0.119
**To what extent you suffer from dry mouth at night?**			
Baseline	85 (40/10)	80 (50/100)	0.026
Follow-Up	80 (40/100)	80 (10/100)	0.258
**How often do you wake up on average at night due to dry mouth?**			
Baseline	2.5 (0/12)	2.5 (0/40)	0.901
Follow-Up	3 (0/7)	3 (0/4.5)	0.067

**Table 4:** Subjective patient-reported outcomes after one week of use: comparison between water and adhesive discs

Mean (SD) Median range	Water (Median; Min/Max)	Adhesive discs (Median; Min/Max)	*p*
How effective do you think the product is in improving dry mouth symptoms after a week of use?	25 (0/95)	62.5 (0/100)	**< 0.001**
How did you like the taste of the product after the week of use?	50 (5/100)	70 (20/100)	**0.017**
How comfortable did your mouth feel after using the product for a week?	50 (10/100)	65.5 (20/100)	**0.004**
After application, did you feel that applying the product to the mucous membrane created a moisturizing effect/film of moisture?	38 (0/90)	75 (0/100)	**< 0.001**
Did you feel that you felt a reduced need to drink at night after the application?	20 (0/100)	70 (0/100)	**0.002**

Abbreviations: SD – Standard Deviation

### Objective measures

Neither treatment condition showed significant within-group changes in salivary function. In the water group, USFR increased slightly from 0.58 to 0.62 ml/5 min (*p* = 0.20), while in the adhesive discs group it rose from 0.70 to 0.90 ml/5 min (*p* = 0.094). SSFR demonstrated minor non-significant decreases in both groups. Dry-mouth VAS scores remained largely unchanged over the one-week period (water: *p* = 0.119; adhesive discs: *p* = 0.509), with no meaningful pre–post differences (Table 5). Nighttime symptom-related disruptions differed between the two study phases. The frequency of nighttime water intake was 0.43 ± 0.69 times/night during the adhesive discs phase and 1.13 ± 1.27 times/night during the water phase.

**Table 5:** Objective measures pre-post adhesive discs versus water

	Water Median (range)	Adhesive discs Median (range)	*p*
**USFR (ml/ 5 min) BL** **USFR (ml/ 5 min) FU** ***p***	0.58 (0.00/3.37)	0.7 (0.00/3.26)	0.051 0.20
	0.62 (0.00/3.36)	0.9 (0.00/4.75)	
	0.150	0. 094	
**SFR (ml/ 5 min) BL** **SFR (ml/ 5 min) FU** ***p***	4.87 (0.12/16.56)	4.45 (0.00/18.15)	0.837 0.458
	4.22 (0.02/17.95)	3.73 (0.05/16.00)	
	0. 586	0. 093	
**VAS BL** **VAS FU** ***p***	70 (20/95)	70 (0/95)	0.579 0.119
	71 (20/99)	70 (10/100)	
	**0.05** ^**+**^	0.509	

Abbreviations: USFR – Unstimulated Salivary Flow Rate; SFR – Stimulated Salivary Flow Rate; VAS – Visual Analogue Scale; BL – Baseline; FU – Follow-up

### Adverse events and tolerability

Adhesive discs were well tolerated overall. Only one participant reported problems with tolerability or side effects to the product. The participant reported a mild tolerability issue, noting that the discs were perceived as too large and caused a temporary sensation of discomfort in the mouth during the first days of use. The incidence of adverse events was low and comparable between groups (Adhesive discs: 3.0%; water: 6.1%; *p* = 0.706). Overall, 98.4% of the participants rated the product as tolerable.

## Discussion

This randomized, crossover-controlled trial aimed to evaluate the short-term efficacy and tolerability of adhesive discs in patients with radiotherapy-induced dry mouth following head and neck cancer (HNC) treatment. Based on the findings, the first null hypothesis must be rejected. Patients reported significant improvements in subjective xerostomia symptoms during the adhesive discs phase compared to water, patient-reported outcome measures and frequency of nocturnal awakenings. Notably, 76% of participants experienced meaningful symptom relief with adhesive discs, and significantly fewer participants reported waking at night due to dry mouth. Conversely, the second null hypothesis cannot be rejected, as no statistically significant changes were observed in USFR or SSFR following either intervention. The marked improvement in subjective symptoms underscores the clinical relevance of adhesive discs as a symptomatic treatment in this vulnerable patient population. Xerostomia following radiotherapy is a persistent and debilitating condition in successfully treated HNC patients, and options for meaningful relief remain limited [16]. This study supports the existing evidence by Burgess and Lee [11], extending their findings with a controlled design and additional endpoints. The present results also align with prior research indicating that subjective xerostomia and objective hyposalivation often do not correlate tightly, especially in patients with irreversible salivary gland damage [17]. Although improvements in perceived oral comfort and dryness are important contributors to patients’ quality of life, the absence of measurable changes in unstimulated or stimulated salivary flow rates restricts the conclusions that can be drawn regarding the overall therapeutic impact of adhesive discs. Because persistent hyposalivation is associated with clinically relevant consequences, such as caries, oral infections, and candidiasis, subjective symptom relief should therefore be interpreted as rather symptomatic benefit [15]. A key finding of this study is the alleviation of nocturnal xerostomia symptoms, which were frequently reported as particularly burdensome [18]. Nocturnal oral dryness is known to disrupt sleep continuity and contribute to fatigue, impaired cognitive function, and reduced quality of life [19]. Importantly, salivary flow cannot be measured during sleep, which underscores the necessity of subjective assessment tools for evaluating nighttime symptom control [20]. The targeted questionnaire on nocturnal awakenings revealed a clinically meaningful benefit of adhesive discs in this context. This observation is of particular interest given that most existing clinical trials and guidelines rarely consider the temporal patterns of xerostomia symptoms, especially nocturnal discomfort. Despite these marked subjective improvements, no statistically significant differences were observed in objective salivary flow parameters. This dissociation between perceived and measured effects is in line with earlier reports on saliva substitutes and topical agents used in irradiated patients [17]. The discrepancy may be attributed to irreversible glandular damage resulting from radiotherapy, where true functional regeneration of salivary tissue is unlikely. Nonetheless, this should not preclude the implementation of therapeutic strategies aimed at improving mucosal lubrication and perceived oral comfort. The mild upward trend in USFR observed during adhesive discs use may suggest a transient stimulatory effect – possibly mediated by mechanical stimulation – but this did not reach statistical significance. These findings align with recent clinical data on other mucoadhesive formulations [21]. In a randomized crossover trial by Iacovelli et al., Aqualief™, a mucoadhesive tablet containing carnosine and karkadé, was shown to stimulate salivary flow and reduce salivary pH drop in HNC patients with radiation-induced xerostomia, compared to placebo) [21]. While the Aqualief™ study focused more on physiological endpoints and had a slightly different compound profile, the positive effects observed in both trials underline the growing relevance of topical, palliative therapies for this patient population.

The perceived benefits of adhesive discs may be attributed to their gradual release of xylitol and cellulose gum, forming a mucoadhesive, lubricating film that mimics the natural properties of saliva [11]. These mechanisms are supported by previous findings showing that texture can have strong effects on oral comfort in xerostomia patients [8]. Notably, detailed evaluation of dichotomous items from the Shahdad questionnaire indicated significant subjective improvements in parameters such as perceived oral moisture, sleep continuity, and coating sensation, especially during nighttime. These improvements are of clinical relevance, as nocturnal xerostomia is often under-addressed yet significantly impairs sleep quality, daytime functioning, and well-being [18, 20].

Tolerability was good, with only one reported case of product intolerance and no significant difference in adverse event rates compared to the control group. The majority of participants rated the product favorably in terms of taste, comfort, and ease of use. These findings are particularly relevant for nighttime application, where product discreteness and long-wearing comfort are critical for adherence [22]. The findings suggest that adhesive discs can offer a practical, low-cost, and well-accepted option for nighttime xerostomia management. In daily clinical practice, they may serve as an adjunctive strategy to improve oral comfort, reduce sleep disruption, and ultimately support the quality of life in HNC survivors, an area where few effective interventions exist. To complement the quantitative findings, selected patient quotes illustrating subjective experiences with adhesive discs are provided in the Supplement S2.

## Limitations

This study has several limitations. The use of water as a control does not represent a true placebo, as it lacks adhesive and lubricating properties and dissipates rapidly in the oral cavity. This limits its ability to mimic the physical characteristics of a placebo disc and may affect the interpretability of the observed treatment effects relative to an inert comparator. Although a one-week washout period was implemented, its sufficiency remains uncertain; however, given the short-acting nature of the intervention, carryover effects are unlikely. Although the specific radiation techniques (e.g., IMRT vs. 3D-CRT, VMAT) could not be reliably retrieved from the available documentation and therefore could not be analyzed separately, this limitation does not compromise the overall interpretability of the study. Comprehensive treatment information, including total radiation dose, number of fractions, treatment duration, and underlying tumor diagnosis, was available for all patients. These parameters provide a robust basis for estimating salivary gland exposure and allow meaningful conclusions regarding treatment-related glandular impairment. The brief duration of the intervention limits conclusions regarding long-term effectiveness, tolerability, and adherence. Furthermore, the relatively small sample size restricts generalizability and may reduce statistical power, particularly for detecting subtle changes in objective parameters. The reliance on subjective outcome measures introduces potential bias due to intraindividual variability and the absence of objective markers for mucosal hydration or microbial changes. In light of the present findings, future studies should aim to validate the clinical benefits of adhesive discs in larger, multicenter cohorts with extended intervention periods and standardized assessment protocols. Incorporating additional parameters, such as microbiological profiling, objective mucosal wetness measurements, and long-term dental outcomes, may provide a more comprehensive understanding of the therapeutic potential of this intervention. To strengthen methodological rigor, future trials should also include a well-designed placebo that mimics the physical characteristics of adhesive discs (e.g., disc form, adhesion, neutral taste) but lacks active ingredients such as xylitol or flavoring agents. This would enable more effective blinding and a clearer differentiation between the effects of mucoadhesive texture, gustatory stimulation, and the specific pharmacological properties of the active components.

## Conclusion

The results of this study suggest that symptom-oriented, topical approaches may contribute to the management of nocturnal xerostomia in patients with long-term sequelae of head and neck radiotherapy. The use of a mucoadhesive disc containing xylitol and cellulose gum was well tolerated and associated with improved subjective oral comfort and reduced nighttime burden. These findings underline the relevance of addressing patient-reported outcomes in supportive care strategies, particularly when functional regeneration of salivary gland tissue is no longer achievable. While the observed effects were limited to subjective domains, the consistency of reported benefits warrants further investigation in larger, more diverse populations and over longer timeframes.

## Supplementary Information

Below is the link to the electronic supplementary material.

Supplementary Material 1

Supplementary Material 2

## Data Availability

No datasets were generated or analysed during the current study.

## Abbreviations

API: Approximal Plaque Index
SD: Standard Deviation
VAS: Visual Analogue Scale
pH: Potential of Hydrogen (Acidity level)
