# Supplementary material for: Efficacy of adhesive discs for nocturnal xerostomia after head and neck radiotherapy: a randomized crossover trial
Source: Clin Oral Investig. 2026 Feb 28;30(3):101. doi: 10.1007/s00784-026-06798-8 (PMC12948901; doi:10.1007/s00784-026-06798-8)
Supplement: Supplementary file 1 — Supplementary Material 1 [file 784_2026_6798_MOESM1_ESM.docx]

**Supplementary Table S1: Overview of Diagnoses, Tumor Staging, and Radiotherapy Parameters**

| **Diagnosis** | **Tumor staging** | **Radiation period (weeks)** | **Number of fractions** | **Total dose (Gy)** |
| --- | --- | --- | --- | --- |
| Base of tongue carcinoma | cT4a cN2b cM0 | 6 | 30 | 60 |
| Base of tongue carcinoma | cT4 cN0 cM0 | 6 | 33 | 68.6 |
| Lateral tongue carcinoma | cT2 cN0 cM0 | 6 | 30 | 60 |
| Tonsillar carcinoma | pT1 pN1 cM0 | 12 | 37 | 172.8 |
| Hodgkin’s disease | NA | 2 | 10 | 20 |
| Oropharyngeal carcinoma | cT4 cN2 cM0 | 8 | 33 | 68.6 |
| Base of tongue carcinoma | cT4 cN1 cM0 | 7 | 33 | 68.6 |
| Tonsillar carcinoma | pT2 pN3b M0 | NA | NA | 60 |
| Base of tongue carcinoma | pT1 pN2c cM0 | 6 | 29 | 58 |
| Lateral tongue carcinoma | pT2 pN0 cM0 | NA | 30 | NA |
| Adenoid cystic carcinoma | pT1 cN0 Mx | 8 | 40 | 72 |
| Tonsillar carcinoma | pT2 pN1 M0 | 7 | 30 | 64 |
| Nasopharyngeal carcinoma | cT1 cN0 cMx | 7 | 33 | 68.6 |
| Oropharyngeal carcinoma | cT3 cN2c cM0 | 6 | 31 | 72 |
| Cervical CUP syndrome | cTx pN1 cM0 | 6 | 33 | 66 |
| Cervical CUP syndrome | cTx cN3b cM0 | 6 | 30 | 60 |
| Cervical CUP syndrome | cTx pN1 cM0 | 6 | 30 | 60 |
| Oropharyngeal carcinoma | cT2 cN1 cM0 | 6 | 30 | 60 |
| Tonsillar carcinoma | pT3 pN1 cM0 | 7 | 30 | 60 |
| Tonsillar carcinoma | cT4b cN2b cM0 | 6 | 32 | 68 |
| Lateral tongue carcinoma | pT1 cN0 M0 | NA | NA | 70.2 |
| Plasmacytoma of oropharyngeal wall | cT2 cNx cMx | 5 | 23 | 46 |
| Oropharyngeal carcinoma | cT3 cN2 cM0 | 7 | 33 | 68.3 |
| Cervical CUP syndrome | cTx pN1 cM0 | 6 | 30 | 60 |
| Hypopharyngeal carcinoma | pT2 pN2a pMx | 8 | 34 | 59.4 |
| Nasal cavity squamous carcinoma | pT1 cN0 cM0 | NA | NA | 60 |
| Nasopharyngeal carcinoma | cT3 cN3 M0 | 7 | 33 | 68.6 |
| Mediastinal lymph node metastases | pTx pN2b M0 | 12 | 60 | 108 |
| Base of tongue carcinoma | pT1 pN2b cM0 | 6 | 30 | 60 |
| Oropharyngeal carcinoma | cT3 cN1 M0 | 6 | 30 | 60 |
| Oropharyngeal carcinoma | cT4 cN2 cM0 | 6 | 32 | 68.6 |
| Tonsillar carcinoma | cT1 cN0 M0 | 12 | 60 | 108 |
| SCC alveolar mucosa mandible | pT2 pN2 | 6 | 30 | 60 |

Abbreviations**:** CUP, cancer of unknown primary; Gy, Gray; Mx, distant metastasis cannot be assessed; NA, not available; pT/pN/cT/cN/cM, pathological/clinical tumor–node–metastasis classification; SCC, squamous cell carcinoma.
