# Supplementary material for: Efficacy of adhesive discs for nocturnal xerostomia after head and neck radiotherapy: a randomized crossover trial
Source: Clin Oral Investig. 2026 Feb 28;30(3):101. doi: 10.1007/s00784-026-06798-8 (PMC12948901; doi:10.1007/s00784-026-06798-8)
Supplement: Supplementary file 2 — Supplementary Material 2 [file 784_2026_6798_MOESM2_ESM.docx]

Supplement S2. To complement the quantitative results, qualitative insights were gathered from patient statements during feedback documentation. These original comments provide a deeper understanding of the perceived benefits and limitations of XyliMelts beyond standardized scales:

*Patient 1:
“With XyliMelts, my mouth stayed noticeably more moist during the night, and I no longer had to get up to drink.”
“My swallowing improved significantly, especially at night.”
“Chewing gum helps quickly during the day, but XyliMelts lasts longer and doesn’t irritate the mucosa.”
Patient 2:
“XyliMelts helps me during sports – even with heavy breathing, my mouth no longer feels as dry.”
“The disc adheres reliably, even during sudden movements like in tennis.”
“At night, I use one or two tablets, depending on how dry my mouth feels.”*

*Patient 3:
“With XyliMelts, I sleep better – it creates a coating in the mouth that lasts through the night.”
“The dryness at night is significantly improved. I feel more refreshed in the morning.”*

*Patient 4:
“In the beginning, the tablets felt too large and were a bit bothersome.”
“After a while, they became mushy, which was unpleasant.”*
